# CHRNA5 Contributes to Hepatocellular Carcinoma Progression by Regulating YAP Activity

**DOI:** 10.3390/pharmaceutics14020275

**Published:** 2022-01-25

**Authors:** Yan Fu, Hongfei Ci, Wei Du, Qiongzhu Dong, Huliang Jia

**Affiliations:** 1 Department of General Surgery, Huashan Hospital, Fudan University, Shanghai 200040, China; 19111220072@fudan.edu.cn (Y.F.); 20211220051@fudan.edu.cn (H.C.); 21211220043@fudan.edu.cn (W.D.); 2 Key Laboratory of Whole-Period Monitoring and Precise Intervention of Digestive Cancer, Shanghai Municipal Health Commission (SMHC), Institute of Fudan-Minhang Academic Health System, Minhang Hospital, Fudan University, Shanghai 200437, China

**Keywords:** HCC, CHRNA5, metastasis, stemness property, sorafenib sensitivity

## Abstract

Hepatocellular carcinoma (HCC) is a major health concern worldwide. A better understanding of the mechanisms underlying the malignant phenotype is necessary for developing novel therapeutic strategies for HCC. Signaling pathways initiated by neurotransmitter receptors, such as α5-nicotinic acetylcholine receptor (CHRNA5), have been reported to be implicated in tumor progression. However, the functional mechanism of CHRNA5 in HCC remains unclear. In this study, we explored the role of CHRNA5 in HCC and found that CHRNA5 expression was increased in human HCC tissues and positively correlated with the T stage (*p* < 0.05) and AJCC phase (*p* < 0.05). The KM plotter database showed that the high expression level of CHRNA5 was strongly associated with worse survival in HCC patients. Both in vitro and in vivo assays showed that CHRNA5 regulates the proliferation ability of HCC by regulating YAP activity. In addition, CHRNA5 promotes the stemness of HCC by regulating stemness-associated genes, such as Nanog, Sox2 and OCT4. Cell migration and invasion assays demonstrated that CHRNA5 significantly enhanced the metastasis of HCC by regulating epithelial–mesenchymal transition (EMT)-associated genes. Furthermore, we found that CHRNA5 regulates the sensitivity of sorafenib in HCC. Our findings suggest that CHRNA5 plays a key role in the progression and drug resistance of HCC, and targeting CHRNA5 may be a strategy for the treatment of HCC.

## 1. Introduction

HCC, one of the most common fatal tumors with an increasing incidence rate, is the fourth most common cause of cancer-related death worldwide [1]. Various factors, including chronic hepatitis B virus (HBV) infection, alcohol consumption, and type 2 diabetes, are regarded as triggers for HCC development [2,3]. Although great efforts have been made in developing innovative therapeutic strategies for HCC, the five-year survival rate remains low, mainly due to the high rates of drug resistance, tumor metastasis, and recurrence [4]. Sorafenib is a well-known treatment agent for HCC. By targeting several tyrosine kinases, such as VEGFR, PDGFR, and RAF, sorafenib can suppress the proliferation and angiogenesis of tumors [5]. However, limited benefits were observed in HCC patients receiving sorafenib treatment, mainly due to the development of drug resistance [6]. HCC cells that survived long-term treatment with sorafenib exhibited enhanced stemness properties and an EMT phenotype, which were closely associated with the resistance of various anticancer therapies and cancer remission [7]. Thus, deciphering the mechanisms underlying stemness properties and sorafenib resistance is essential for HCC treatment.

The Hippo signaling pathway plays a crucial role in regulating organ development, tissue hemostasis, and regeneration [8]. Dysregulation of the Hippo signaling pathway has already been observed in multiple tumors, including HCC [9]. Yes-associated protein (YAP) and tafazzin (TAZ), two core transcriptional coactivators in the Hippo signaling pathway, are regulated by a series of kinase cascades consisting of the serine/threonine kinases mammalian sterile 20-like kinase 1 and 2 (MST1 and MST2) and large tumor suppressor 1 and 2 (LATS1 and LATS2) [10]. YAP and TAZ were retained in the cytoplasm and deprived of transcriptional activity after being phosphorylated by LATS1/2. Inactivation of the Hippo signaling pathway leads to increased YAP activity, contributing to the enhanced malignant phenotype of tumor cells. In HCC, Hippo signaling can inhibit HCC formation, and activation of YAP transcription activity is critical for HCC proliferation [11]. The contribution of YAP to drug resistance in HCC was also reported, and manipulating YAP activity may be a plausible therapeutic strategy for HCC.

Nicotinic acetylcholine receptors (nAChRs), ligand-gated ion channels that are mainly expressed in the plasma membranes of certain neurons on the postsynaptic side of the neuromuscular junction, are also expressed in some non-neuronal cells [12]. nAChRs can be activated to mediate fast signal transmission at synapses by the endogenous neurotransmitter acetylcholine (ACh) or by the exogenous tertiary alkaloids nicotine and tobacco alkaloid [13,14,15]. nAChR-based targeted therapies for nervous system disorders, including Alzheimer’s disease, depression, Parkinson’s disease, Tourette’s syndrome, and nicotine addiction, have been investigated [15,16]. Recently, numerous studies revealed that nAChRs also play significant roles in cancer progression [17]. For example, α7-nAChR was found to be associated with cancer cell proliferation and migration, exhibiting the potential to serve as a therapeutic target for tumors [18]. α5-Nicotinic acetylcholine receptor (CHRNA5) is a member of the nicotinic acetylcholine receptor superfamily. CHRNA5 was initially recognized as an important regulator in nicotine addiction and nicotine-dependent lung cancer development [19,20]. CHRNA5-mediated Ca^2+^ influx was found to activate MAPK and VEGF signaling pathways, thereby contributing to tumor progression in lung cancer [21]. Recently, several studies identified the critical role of CHRNA5 in several other cancers. CHRNA5, upregulated in breast cancer, was identified as the secondary estrogen signaling network and exhibited prognostic value in breast cancer [22]. In melanoma, CHRNA5 was reported to modulate cancer growth by regulating the Notch1 signaling pathway [23]. Another study found that CHRNA5 could promote radioresistance via regulating E2F transcription factor activity in oral squamous cell carcinoma [24]. However, the role of CHRNA5 in HCC remains largely unknown. Here, our study focused on revealing the role of CHRNA5 in HCC progression. We found that CHRNA5 contributes to HCC progression through the YAP-dependent modulation of proliferation ability, the EMT phenotype, and stemness properties. Our study demonstrates the clinical and biological significance of CHRNA5 in HCC, and CHRNA5 might serve as a promising prognostic biomarker and therapeutic target for HCC.

## 2. Materials and Methods

### 2.1. Cell Culture

Human HCC cell lines Huh7 and PLC/PRF/5 (PLC) were obtained from Shanghai Cell Bank, Chinese Academy of Sciences. All cell lines were cultured in Dulbecco’s modified Eagle’s medium (DMEM, HyClone, Logan, UT, USA) supplemented with 10% fetal bovine serum (FBS; Gibco, Carlsbad, CA, USA) and penicillin/streptomycin (1%; Gibco, Carlsbad, CA, USA). Cells were maintained in a humidified incubator with 5% CO_2_ at 37 °C. 

### 2.2. Patient Samples

In total, 70 paired HCC samples and adjacent normal counterparts were obtained from Huashan Hospital from November 2015 to December 2016. Diagnoses were made by two pathologists independently. All specimens were fixed with formalin and embedded in paraffin. Written informed consent was provided by all patients. The methods and experimental protocols performed in this study were approved by the Human Research Ethics Committee of Huashan Hospital.

### 2.3. RNA Interference and Plasmid Transfection

When the cells reached 70% confluence, transfection was carried out according to the lipofectamine 3000 (Invitrogen, Waltham, MA, USA) instructions. Lentiviral short hairpin RNA (shRNA) targeting CHRNA5 (CCGGGCTCGATTCTATTCGCTACATCTCGAGATGTAGCGAATAGAATCG AGCTTTTTG for sh1, and CCGGCCTGATGACTATGGTGGAATACTCGAGTATTCCACCATAGTCATCA GGTTTTTG for sh2) and control vectors (sh-NC) obtained from Sigma-Aldrich were loaded into the PLKO plasmid. The CHRNA5 CDS sequence was loaded into the PCDH plasmid for CHRNA5 overexpression in HCC cells.

### 2.4. Cell Viability Assay

CCK8 test kits were employed to test cell viability following the manufacturer’s instructions. After being seeded in 96-well plates, cancer cells were incubated with DMEM containing 10% CCK8 for 2 h, and the absorbance was measured at 450 nm.

### 2.5. TdT-Mediated dUTP Nick-End Labeling (TUNEL) Assay

To detect sorafenib-induced apoptosis, we conducted a TUNEL assay according to the manufacturer’s instructions (Beyotime Biotechnology, Shanghai, China). DAPI was used to stain the nucleus. Cells were imaged with a fluorescent microscope.

### 2.6. cDNA Synthesis and qRT-PCR Assay

Trizol reagent (Invitrogen, Waltham, MA, USA) was used to extract total RNA, and NanoDrop2000 was used for RNA quantification. The total RNA was reverse-transcribed into cDNA for qRT-PCR analysis following the manufacturer’s instructions for PrimeScript RT Reagent Kit (TaKaRa, Otsu City, Shiga Prefecture, Japan). Then, quantitative real-time PCR assays were conducted using the ABI7500 system according to the protocol. The primer sequences for qRT-PCR were as follows: CHRNA5-Forward: 5′-GCCAGAGTGCCAGTGAGAAG-3′, CHRNA5-Reverse: 5′-CGAGGCCAGCTGAGCAA-3′; GAPDH-Forward: 5′- TCGGAGTCAACGGATTTGGT-3′, GAPDH-Reverse: 5′- TTCCCGTTCTCAGCCTTGAC-3′.

### 2.7. Western Blot

The protein concentration was determined using a BCA kit after cells were lysed in radioimmunoprecipitation assay (RIPA) buffer. Then, the proteins were boiled with loading buffer at 100 ℃ for 15 min. Polyacrylamide gel electrophoresis and membrane transfer were carried out as previously described [25]. The membrane was incubated with primary antibodies against CHRNA5 (Thermo Fisher Scientific, Waltham, MA, USA), E-cadherin (CST, Danvers, MA, USA), N-cadherin (CST, Danvers, MA, USA), vimentin (CST, Danvers, MA, USA), YAP (CST, Danvers, MA, USA), OCT4 (Abclonal, Wuhan, Hubei, China), Nanog (Abclonal, Wuhan, Hubei, China), Sox2 (Abclonal, Wuhan, Hubei, China), histone H3 (CST, Danvers, MA, USA), and GAPDH (CST, Danvers, MA, USA) at 4 °C overnight. Then, the membrane was further incubated with secondary antibody for one hour at room temperature, and enhanced chemiluminescence (ECL) reagent was used to visualize the protein bands using a Gel Doc EZ Imager. GAPDH was used as an internal reference.

### 2.8. Colony Formation Assay

Transfected Huh7 and PLC cells (1000 cells/well) were seeded into 6-well plates and cultured for 2 weeks. Finally, colonies were counted after being fixed with 4% paraformaldehyde and stained with 0.1% crystal violet.

### 2.9. Cell Migration and Invasion Assay

Cells at the logarithmic growth phase were harvested and resuspended in serum-free medium to be seeded in transwell chambers for detecting their migration ability. For the invasion assay, cells were seeded in transwell chambers containing Matrigel. After 24 h or 48 h, the transwell chambers were collected. Cells were fixed with 4% paraformaldehyde and stained with crystal violet. The numbers of invading and migrating cells were counted under a light microscope.

### 2.10. CHRNA5 Expression and Clinical Information from the Cancer Genome Atlas (TCGA)

The RNA-sequencing-based gene expression data of 374 HCC tumor samples and 50 normal samples were downloaded using the “TCGAbiolinks” package (TCGAbiolinks: an R/Bioconductor package for integrative analysis of TCGA data). The clinical data of the corresponding 374 HCC patients were downloaded from The Cancer Genome Atlas (TCGA, https://portal.gdc.cancer.gov/, last date for accession: 15 August 2021). We used “R” to normalize the original RNA-sequencing data to transcripts per million reads (TPM) [26]. After excluding the samples lacking mRNA expression or clinical information, we included 370 HCC patients for the clinical and prognosis analysis.

### 2.11. Gene Ontology (GO) and Kyoto Encyclopedia of Genes and Genomes (KEGG) Enrichment Analysis

The genes correlated with CHRNA5 were confirmed using “corr” packages in R. To assess the biological function of CHRNA5-correlated genes, Gene Ontology (GO) and Kyoto Encyclopedia of Genes and Genomes (KEGG) pathway analyses were performed using the R package “clusterProfiler”, and values with *p* < 0.05 were considered to be statistically significant [27]. All statistical data analyses in this study were performed using R software (version 4.1.1).

### 2.12. Animal Study

PLC-NC, PLC-sh CHRNA5, Huh7-CMV, and Huh7-CHRNA5 OE cells (2 × 10^6^) were injected subcutaneously into the left flank of 3–4-week-old male nude mice kept in the SPF animal laboratory. Tumor weight was measured after sacrificing the mice by anesthesia at the end of the experiment. Every nude mouse received humane care in accordance with the National Institutes of Health guidelines (NIH Publications No. 8023). The animal study was performed following the protocols approved by the Institutional Animal Care and Use Committee of Fudan University.

### 2.13. Statistical Analysis

For statistical analysis, experiments were repeated three times. All data were analyzed by SPSS 21.0 (Chicago, IL, USA) and GraphPad Prism 8.0.1 (La Jolla, CA, USA). χ^2^-Tests, two-tailed Student’s *t*-test, Spearman’s rank correlation, and Kaplan-Meier analysis were used according to the data type. Values with *p* < 0.05 were regarded as statistically significant.

## 3. Results

### 3.1. CHRNA5 Is Significantly Overexpressed in Hepatocellular Carcinoma and Correlated with Poor Prognosis of HCC Patients

**Figure 1:**
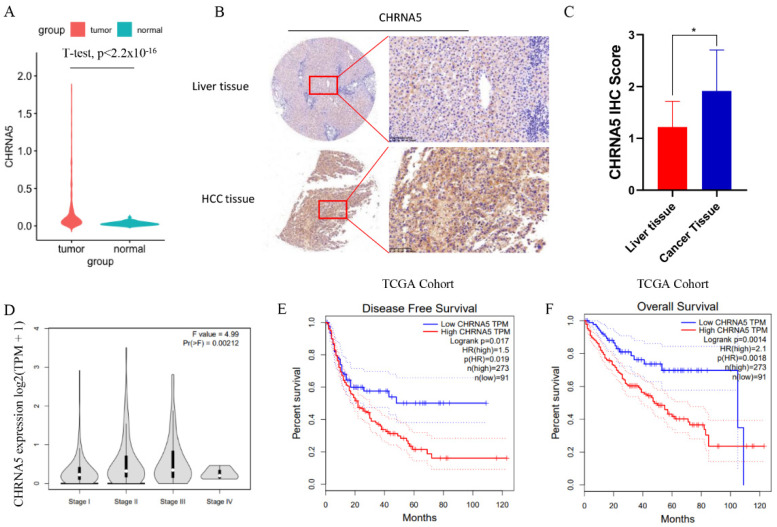
Increased expression of CHRNA5 in HCC. (**A**) Increased mRNA expression of CHRNA5 in HCC compared with that in normal liver tissue; (**B**,**C**) increased protein expression of CHRNA5 in HCC compared with that in normal liver tissue; (**D**) CHRNA5 mRNA expression level in HCC at different stages; (**E**,**F**) relationship between CHRNA5 expression and the prognosis of HCC patients. * *p* < 0.05.

To investigate the expression pattern of CHRNA5 in HCC tissues, we analyzed data from the TCGA database and found that the mRNA expression level of CHRNA5 was significantly higher in HCC tissues compared with that in adjacent normal liver tissues (*p* < 0.05) (Figure 1A). We further detected the CHRNA5 protein level and also found increased CHRNA5 expression in HCC tissues (Figure 1B,C). The correlation between CHRNA5 expression and the corresponding clinicopathological parameters was analyzed using data from the TCGA database. The results suggested that there was a close association between the CHRNA5 mRNA expression level and tumor stage (Figure 1D), suggesting that CHRNA5 plays a role in tumor progression. We further explored the relationship between the CHRNA5 expression level and the clinical characteristics of HCC patients, including gender, age, T stage, distant metastasis, AJCC phase, and vascular invasion. The results revealed significant correlations between the CHRNA5 expression level and T stage (*p* < 0.05) and AJCC phase (*p* < 0.05) (Table 1). Using the GEPIA database to analyze the prognostic value of CHRNA5 in HCC, we found that HCC patients with a higher mRNA expression level of CHRNA5 exhibited poorer disease-free survival and overall survival after surgery (Figure 1E,F). These results indicate that CHRNA5 contributes to cancer progression and poor prognosis in HCC patients. 

### 3.2. CHRNA5 Promotes Proliferation of HCC Cells

**Figure 2:**
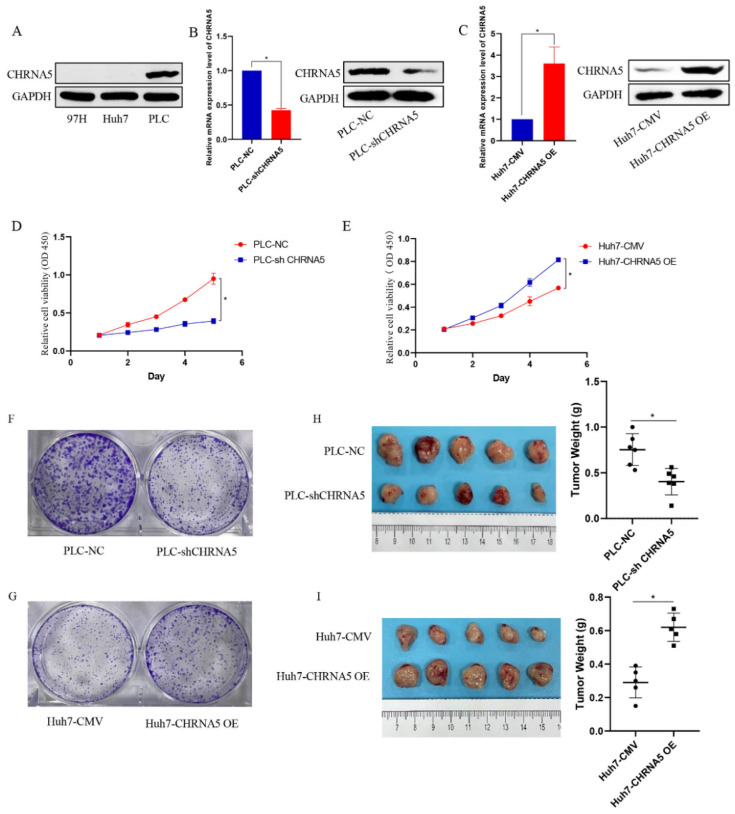
CHRNA5 regulates the proliferation ability of HCC cells. (**A**) The protein expression level of CHRNA5 in HCC cell lines, including MHCC-97H, Huh7, and PLC cells; (**B**) real-time qPCR and Western blot analysis of the knockdown efficiency of CHRNA5 in PLC cell lines; (**C**) real-time qPCR and Western blot analysis of the overexpression efficiency of CHRNA5 in Huh7 cell lines; (**D**) CCK8 assay of PLC-NC and PLC-sh CHRNA5 cell lines; (**E**) CCK8 assay of Huh7-CMV and Huh7-CHRNA5 OE cell lines; (**F**) colony formation assay of PLC-NC and PLC-sh CHRNA5 cell lines; (**G**) colony formation assay of Huh7-CMV and Huh7-CHRNA5 OE cell lines; (**H**) image of tumors from PLC-NC and PLC-sh CHRNA5 cell lines subcutaneously injected into mice and tumor weight; (**I**) image of tumors from Huh7-CMV and Huh7-CHRNA5 OE cell lines subcutaneously injected into mice and tumor weight. * *p* < 0.05.

To explore the cellular functions of CHRNA5 in HCC, we generated a CHRNA5-overexpressing Huh7 cell line and a CHRNA5-silencing PLC cell line based on the basal expression level of CHRNA5 in Huh7, PLC, and 97H (Figure 2A–C). The CCK8 assay revealed that silencing CHRNA5 markedly inhibited the proliferation ability of PLC cells, and CHRNA5 overexpression improved the proliferation ability of Huh7 cells (Figure 2D,E), suggesting that CHRNA5 plays a critical role in promoting HCC cell proliferation. We carried out a colony formation assay to further observe the long-term effect of silencing or overexpressing CHRNA5 on the proliferation of HCC cells. As expected, the number of colonies was significantly reduced after silencing CHRNA5 in the PLC cell line and was markedly increased after CHRNA5 overexpression in the Huh7 cell line (Figure 2F,G). To investigate the growth-promoting effect in vivo, we developed subcutaneous xenograft models of PLC-NC, PLC-shCHRNA5, Huh7-CMV, and Huh-CHRNA5 OE cells in nude mice. The results revealed that CHRNA5 silencing significantly inhibited tumor growth, and CHRNA5 overexpression markedly promoted tumor growth in vivo (Figure 1H,I), further suggesting that CHRNA5 plays a critical role in promoting the proliferation ability of HCC cells.

### 3.3. CHRNA5 Promotes Invasion and Migration of HCC

**Figure 3:**
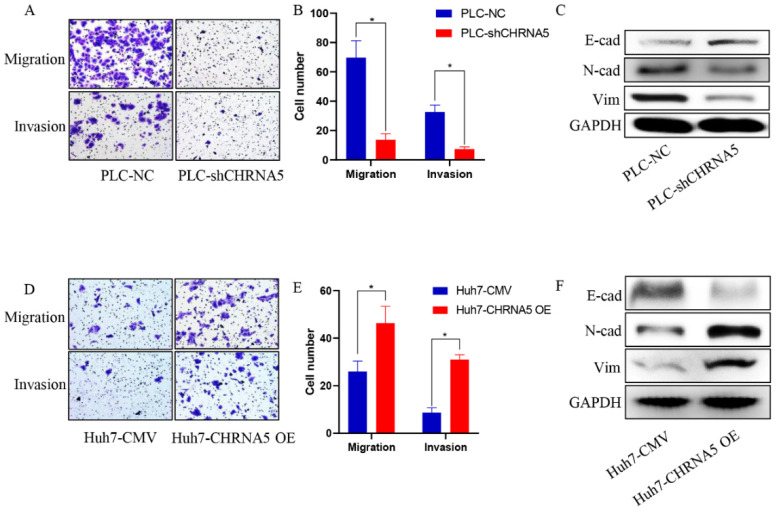
CHRNA5 promotes HCC migration and invasion via regulating EMT phenotype. (**A**,**B**) Effects of silencing CHRNA5 on HCC migration and invasion were determined by transwell assays; (**C**) effects of silencing CHRNA5 on expression of EMT-associated markers in HCC; (**D**,**E**) effects of CHRNA5 overexpression on HCC migration and invasion were determined by transwell assays; (**F**) effects of CHRNA5 overexpression on expression of EMT-associated markers in HCC. * *p* < 0.05.

Furthermore, silencing CHRNA5 markedly inhibited the migration and invasion ability of PLC cells (Figure 3A,B) and downregulated the expression of EMT-associated markers, such as N-cad and vimentin (Figure 3C). Accordingly, CHRNA5 overexpression significantly increased the migration and invasion ability of Huh7 cells by upregulating EMT-associated genes (Figure 3D–F). These results revealed that CHRNA5 was also involved in the migration and invasion of HCC cells by regulating the EMT phenotype.

### 3.4. CHRNA5 Promotes Stemness Properties of HCC

**Figure 4:**
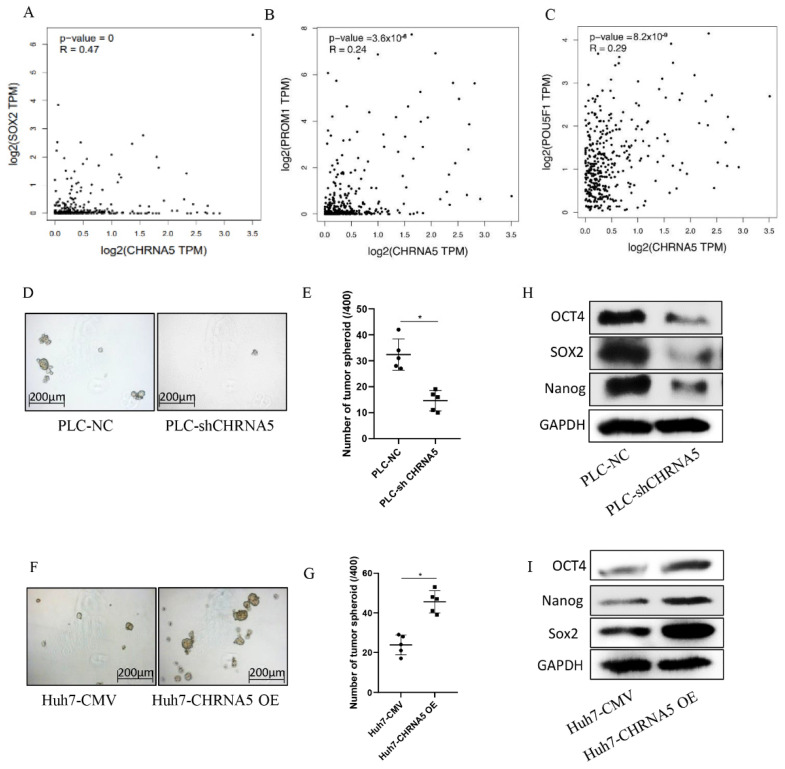
CHRNA5 promotes HCC spheroid formation ability via regulating stemness-associated genes. (**A**–**C**) Correlation between CHRNA5 mRNA expression level and stemness-associated genes in TCGA database; (**D**,**E**) effects of CHRNA5 silencing on HCC spheroid formation ability; (**F**,**G**) effects of CHRNA5 overexpression on HCC spheroid formation ability; (**H**) effects of CHRNA5 silencing on the expression of stemness-associated genes; (**I**) effects of CHRNA5 overexpression on the expression of stemness-associated genes. * *p* < 0.05.

From the TCGA database, we observed that CHRNA5 was positively correlated with stemness-associated genes, such as Sox2, CD133, and OCT4 (Figure 4A–C). The tumor spheroid formation assay is a commonly used method to detect the stemness properties of cancer cells [25]. To further determine whether CHRNA5 plays a regulatory role in maintaining the stemness properties of HCC, we carried out a spheroid formation assay. The results showed that silencing CHRNA5 inhibited the tumor spheroid formation ability of PLC cells (Figure 4D,E), and CHRNA5 overexpression increased the tumor sphere formation ability of Huh7 cells (Figure 4F,G), indicating that CHRNA5 has a regulatory role in maintaining the stemness properties of HCC cells. In addition, Western blot analysis revealed that CHRNA5 participated in regulating stemness-associated genes, such as OCT4, Nanog, and Sox2 (Figure 4H,I). These results indicate that CHRNA5 maintains stemness properties by regulating stemness-associated genes in HCC.

### 3.5. CHRNA5 Regulates YAP Activity in HCC

**Figure 5:**
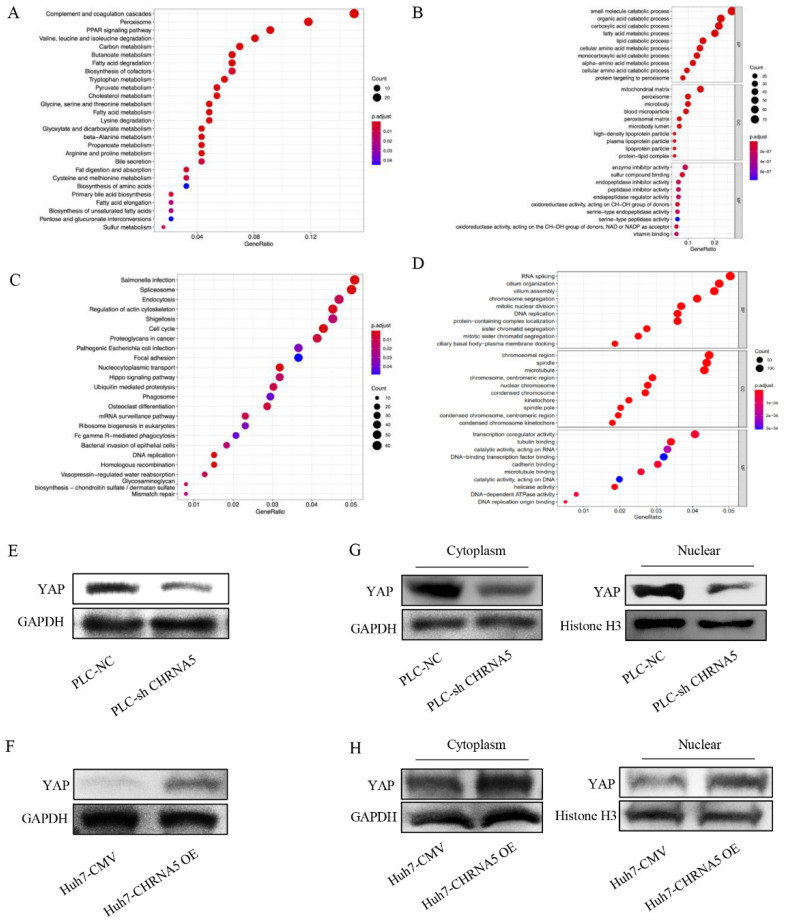
CHRNA5 regulates YAP activity in HCC. (**A**) KEGG analysis of genes negatively correlated with CHRNA5 in HCC; (**B**) GO analysis of genes negatively correlated with CHRNA5 in HCC; (**C**) KEGG analysis of genes positively correlated with CHRNA5 in HCC; (**D**) GO analysis of genes positively correlated with CHRNA5 in HCC; (**E**) Western blot analysis of YAP in PLC-NC and PLC-sh CHRNA5 cell lines; (**F**) Western blot analysis of YAP in Huh7-CMV and Huh7-CHRNA5 OE cell lines; (**G**) Western blot analysis of YAP expression in cytoplasm and nucleus from PLC-NC and PLC-sh CHRNA5 cell lines; (**H**) Western blot analysis of YAP expression in cytoplasm and nucleus from Huh7-CMV and Huh7-CHRNA5 OE cell lines.

We used the online analysis tool LinkedOmics to identify genes that were significantly correlated with CHRNA5 in HCC. Genes negatively correlated with CHRNA5 (Figure 5A,B) and positively correlated with CHRNA5 (Figure 5C,D) were incorporated into KEGG and GO analyses. The results reveal that CHRNA5 is associated with many important cancer-related signaling pathways, such as cell cycle regulation, DNA replication, mismatch repair, and the Hippo signaling pathway in HCC. YAP is an important component of the Hippo signaling pathway and plays a critical role in pro-proliferation and anti-apoptosis [28]. To further check whether CHRNA5 can regulate the Hippo signaling pathway in HCC, we detected the YAP expression level after silencing or overexpressing CHRNA5. The results revealed that YAP expression was significantly downregulated after silencing CHRNA5 and markedly upregulated after overexpressing CHRNA5 in HCC cells (Figure 5E,F). The transcriptional activity of YAP was closely associated with its subcellular localization. Therefore, we further detected the YAP expression level in the cytoplasm and nucleus after CHRNA5 knockdown or overexpression. The results revealed that silencing CHRNA5 significantly inhibited the nuclear accumulation of YAP in the PLC cell line, and CHRNA5 overexpression markedly augmented YAP nuclear accumulation in the Huh7 cell line (Figure 5G,H), further suggesting that CHRNA5 has a critical role in regulating YAP activity.

### 3.6. YAP Plays an Essential Role in the Contribution of CHRNA5 to Malignant Phenotype of HCC

**Figure 6:**
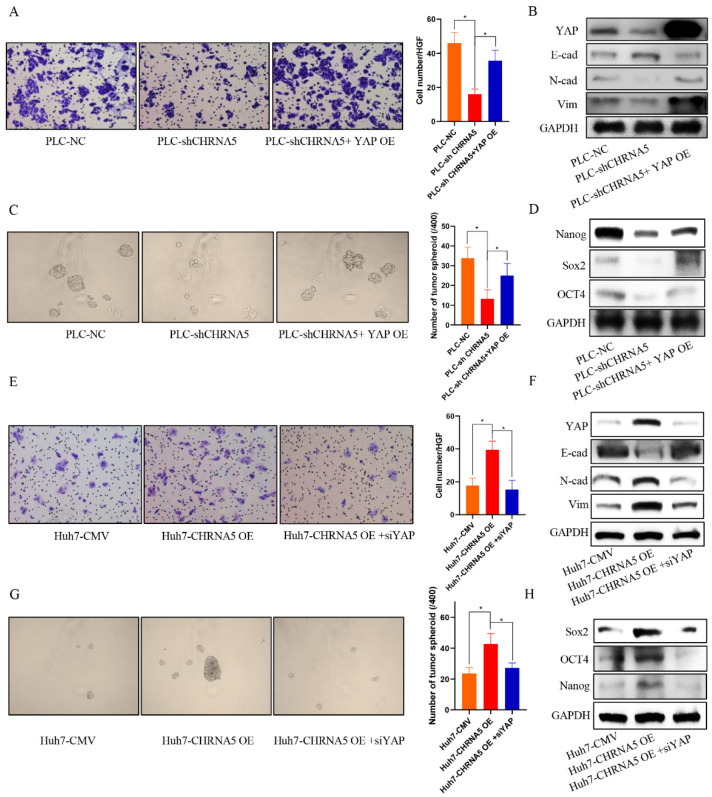
YAP plays an essential role in the contribution of CHRNA5 to malignant phenotype of HCC. (**A**) YAP overexpression partially rescued the inhibitory effect of silencing CHRNA5 on the migration ability of HCC cells; (**B**) YAP overexpression partially rescued the inhibitory effect of silencing CHRNA5 on the expression of EMT-associated markers; (**C**) YAP overexpression partially rescued the inhibitory effect of silencing CHRNA5 on the spheroid formation ability of HCC cells; (**D**) YAP overexpression partially rescued the inhibitory effect of silencing CHRNA5 on the expression of stemness-associated genes; (**E**) the inhibitory effect of silencing CHRNA5 on the migration ability of HCC cells was attenuated after overexpressing YAP; (**F**) the inhibitory effect of silencing CHRNA5 on the expression of EMT-associated markers in HCC cells was attenuated after overexpressing YAP; (**G**) the inhibitory effect of silencing CHRNA5 on the spheroid formation ability of HCC cells was attenuated after overexpressing YAP; (**H**) the inhibitory effect of silencing CHRNA5 on the expression of stemness-associated genes in HCC cells was attenuated after overexpressing YAP. * *p* < 0.05.

To uncover whether YAP plays an essential role in the contribution of CHRNA5 to the HCC malignant phenotype, we overexpressed YAP in CHRNA5-silencing PLC cell lines and silenced YAP expression in CHRNA5-overexpressing Huh7 cell lines. The results revealed that YAP overexpression partially reversed the inhibitory effect of silencing CHRNA5 on the migration ability and expression of EMT-associated markers (Figure 6A,B). Furthermore, the inhibitory effects of silencing CHRNA5 on the tumor spheroid formation ability of PLC cell lines and the expression of stemness-associated genes were also attenuated after overexpressing YAP (Figure 6C,D). Accordingly, the opposite effect was observed after silencing YAP in CHRNA5-overexpressing Huh7 cell lines (Figure 6E–H). These results suggest that CHRNA5 can regulate the malignant phenotype of HCC cell lines by modulating YAP activity.

### 3.7. CHRNA5 Regulates Sorafenib Sensitivity in HCC

**Figure 7:**
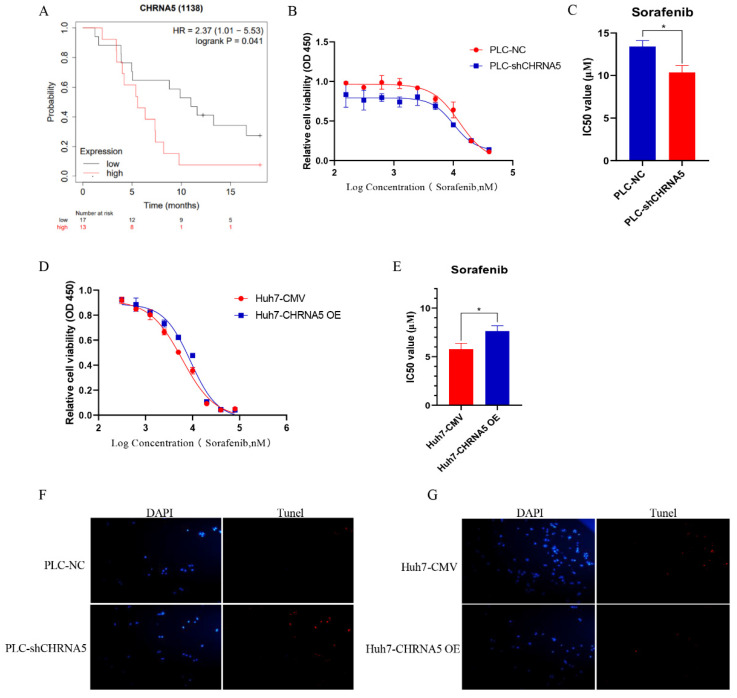
CHRNA5 regulates sorafenib sensitivity in HCC. (**A**) The effect of CHRNA5 on the OS of HCC patients receiving sorafenib treatment; (**B**,**C**) IC50 of sorafenib in PLC-NC and PLC-sh-CHRNA5 cell lines; (**D**,**E**) IC50 of sorafenib in Huh7-CMV and Huh7-CHRNA5 OE cell lines; (**F**) TUNEL detection of PLC-NC and PLC-shCHRNA5 cell lines treated with sorafenib; (**G**) TUNEL detection of Huh7-CMV and Huh7-CHRNA5 OE cell lines treated with sorafenib. * *p* < 0.05, ^+^ Censored Data.

Several studies have already uncovered that YAP activity is closely associated with sorafenib resistance in HCC [29,30]. In addition, stemness properties were also reported to share a close association with sorafenib sensitivity in HCC [31]. Therefore, we further evaluated the effect of CHRNA5 on HCC sensitivity to sorafenib. From the TCGA database, we observed that a higher expression level of CHRNA5 was correlated with a worse OS in HCC patients receiving sorafenib treatment (Figure 7A), suggesting that CHRNA5 might be associated with sorafenib sensitivity in HCC. An in vitro assay also revealed that silencing CHRNA5 significantly decreased the IC50 of HCC cells (Figure 7B,C), which was markedly increased when CHRNA5 was overexpressed (Figure 7D,E). The TUNEL assay also revealed that CHRNA5 silencing augmented the apoptosis of HCC cells induced by sorafenib, whereas CHRAN5 overexpression attenuated the apoptosis of HCC cells induced by sorafenib (Figure 7F,G). These results suggest that CHRNA5 contributes to sorafenib resistance in HCC and has the potential to serve as an indicator for predicting sorafenib sensitivity in HCC patients.

## 4. Discussion

CHRNA5, a member of the superfamily of ligand-gated ion channels, is a vital receptor for nicotine [32]. Thus, previous studies regarding CHRNA5 have largely focused on its role in nicotine dependence and lung cancer progression [33]. In this study, for the first time, we investigated the role of CHRNA5 in HCC. The results reveal that CHRNA5 expression level is upregulated in HCC tissues and is closely associated with tumor T stage, AJCC phase, and patient prognosis. These results suggest the involvement of CHRNA5 in HCC progression. The role of CHRNA5 in tumor proliferation was previously reported. In oral squamous cell carcinoma, CHRNA5 was reported to regulate the activity of the E2F signaling pathway, a critical regulator of cell cycle and stemness properties (33986804), contributing to tumor growth and treatment resistance [24,25]. Ozlen Konu et al. also reported that CHRNA5 silencing in breast cancer significantly inhibited tumor growth, which might be attributed to the significant inhibition of cell-cycle-associated genes after CHRNA5 silencing [34]. Their study suggested that CHRNA5 might regulate cell cycle arrest during the G1 phase by modulating the activity of retinoblastoma family proteins (RB), which regulate E2F1 activity by binding with E2F1 [35]. To further validate the functional role of CHRNA5 in HCC, we induced lentivirus-mediated overexpression or silencing of CHRNA5. We found that CHRNA5 is crucial for the proliferation of HCC cells both in vitro and in vivo, consistent with the conclusion of previous studies in other types of cancers. Tumor invasion and metastasis are challenges in the clinical treatment of HCC. EMT is regarded as a critical step in tumor invasion and metastasis [36]. A previous study suggested that CHRNA5 could regulate the migration and invasion ability of melanoma cells [23]. Similarly, our study indicated that CHRNA5 could enhance the invasion and metastasis ability of HCC by regulating EMT-associated genes. All of these data suggest that CHRNA5 plays a critical role in the malignant phenotype of HCC.

Sorafenib is a multitarget molecular drug and is one of the main treatment strategies for advanced HCC. By suppressing the activity of various receptor tyrosine kinase and VEGF/Raf/MER/ERK-mediated multiple signaling pathways, sorafenib can inhibit tumor cell proliferation and angiogenesis in vivo [37]. Despite the fact that sorafenib can extend the patient’s overall survival, limited benefits were observed in more than 70% of patients with advanced HCC as a result of drug resistance. Therefore, it is clinically meaningful to explore the mechanisms of sorafenib resistance in HCC patients. Our results revealed that CHRNA5 silencing could augment sorafenib sensitivity, and CHRNA5 overexpression could attenuate sorafenib sensitivity, indicating the regulatory role of CHRNA5 in sorafenib sensitivity in HCC. Accordingly, among patients receiving sorafenib treatment, those with a higher expression level of CHRNA5 exhibited a worse OS compared with those with a lower expression level of CHRNA5. These results indicate that CHRNA5 has a pivotal role in sorafenib resistance in HCC. There is a growing amount of evidence indicating that stemness properties, closely associated with cancer initiation and progression, also play a significant role in treatment resistance [38]. The maintenance of stemness properties relies on the activation of stemness-associated genes, such as Sox2, OCT4, and Nanog [38]. Moreover, several stemness-associated signaling pathways, such as Wnt/β-catenin signaling, Notch signaling, and JAK/STAT signaling, also greatly contribute to stemness properties in HCC [39]. In HCC, stemness properties are closely associated with drug resistance, tumor metastasis, and recurrence [39]. Stemness properties are closely associated with the EMT phenotype, which was previously reported to contribute to sorafenib resistance in HCC [40,41]. Several other studies also reported that stemness properties are closely associated with sorafenib resistance in HCC [42]. Ozlen Konu et al. observed a close association between CHRNA5 and Wnt/β-catenin signaling, from which we can propose that CHRNA5 might also regulate the stemness properties of breast cancer cells [34]. Consistent with their study, our study also indicated that CHRNA5 could maintain the stemness properties of HCC cells by regulating stemness-associated genes, to which the regulatory role of CHRNA5 in sorafenib sensitivity could be partially attributed. Stemness properties are also closely associated with chemotherapy resistance and radiotherapy resistance [43]. Thus, it is possible that CHRNA5 might also contribute to resistance to chemotherapy and radiotherapy in HCC, but further experiments are needed to test this hypothesis. In fact, previous studies have already identified the role of CHRNA5 in the resistance to chemotherapy in breast cancer and the resistance to radiotherapy in oral squamous cell carcinoma [24].

YAP, playing an essential role in cancer progression, was reported to be implicated in modulating various malignant phenotypes of cancer cells, such as cell proliferation, migration, invasion, apoptosis resistance, EMT phenotype, and stemness properties [44,45,46]. In this study, data from the TCGA database exhibited a close association between CHRNA5 and the Hippo signaling pathway in HCC. Further in vitro assays revealed that CHRNA5 could regulate YAP activity, and YAP silencing was sufficient to reverse the CHRNA5-mediated tumor-promoting effect. Thus, these results suggest that CHRNA5 promotes HCC progression by modulating YAP activity. Recent studies also suggested the contribution of YAP to drug sensitivity in HCC through various mechanisms. Yuan Zhou et al. reported that YAP contributes to chemotherapy resistance via regulating the Rac family small guanosine triphosphatase 1 (RAC1)—reactive oxygen species (ROS)—mTOR signaling pathway [47]. Darko Castven et al. reported that YAP induced stem-like properties and accounted for the acquired resistance to sorafenib in HCC cells [48]. Ruize Gao et al. also reported that YAP could transcriptionally initiate the expression of SLC7A11, a key transporter maintaining intracellular glutathione homeostasis, causing resistance to sorafenib-induced ferroptosis in HCC [29]. Consistent with previous studies, our study reveals that downregulation of YAP activity caused by CHRNA5 silencing leads to enhanced sensitivity to sorafenib. Thus, YAP plays a critical role in the contribution of CHRNA5 to stemness properties, EMT phenotype, and sorafenib resistance in HCC.

## 5. Conclusions

In conclusion, our study demonstrates that CHRNA5 plays a significant role in promoting the malignant phenotype of HCC by regulating YAP activity. Its role in sorafenib resistance suggests the potential of CHRNA5 to serve as an indicator for sorafenib sensitivity, and targeting CHRNA5 might be a strategy for HCC treatment.

## Figures and Tables

**Table 1:** The association between CHRNA5 mRNA expression and clinicopathological characteristics in HCC.

Characteristics	CHRNA5 Expression		*p* Value
	High	Low	
Age			
≤65	66	65	0.7963
>65	34	38	
Gender			
Male	57	78	0.0072
Female	43	25	
T stage			
T1	46	68	0.0074
T2-4	54	35	
Metastasis			
M0	97	102	0.3639
M1	3	1	
AJCC phase			
I	46	66	0.0112
II–IV	54	37	
Vascular Invasion			
No	63	73	0.296
Yes	37	30	

## Data Availability

No additional data are available.
